# Quinidine: an "Endangered Species" Drug Appropriate for Management of Electrical Storm in Brugada Syndrome

**DOI:** 10.1016/s0972-6292(16)30670-2

**Published:** 2013-09-01

**Authors:** Stelios Paraskevaidis, Efstratios K Theofilogiannakos, Vasileios Kamperidis, Yiannis S Chatzizisis, Konstantinos Tsilonis, Vassilios P Vassilikos, George Dakos, George Stavropoulos, Antonios Ziakas, Stavros Hadjimiltiades, Ioannis H Styliadis

**Affiliations:** 1st Cardiology Department, AHEPA University Hospital, Aristotle University Medical School, Thessaloniki, Greece

**Keywords:** Brugada syndrome, electrical storm, quinidine

## Abstract

Brugada syndrome is an inherited channelopathy associated with an increased risk of syncope and sudden cardiac death. In rare cases it can be manifested with electrical storm. We report two cases of Brugada syndrome that presented with electrical storm and were treated successfully with oral quinidine, an "endangered species" drug.

## Introduction

Brugada syndrome (BS) is an inherited channelopathy characterized electrocardiographically by pseudo-right bundle branch block pattern and ST segment elevation in precordial leads V1-3 [1]. The clinical manifestation of BS varies from asymptomatic form to sudden cardiac death [1]. We report two cases of BS that were presented with electrical storm.

## Case 1

A 38-year-old man with BS, who was treated with ICD implantation two years ago following an episode of syncope and a subsequent positive procainamide test, presented to our emergency department with electrical storm (i.e. three episodes of ventricular tachycardia in the same day) provoking appropriate shocks from the ICD. Since the ICD implantation the patient was under no medication and remained electrically stable and free of any ICD therapy.

**Figure 1:**
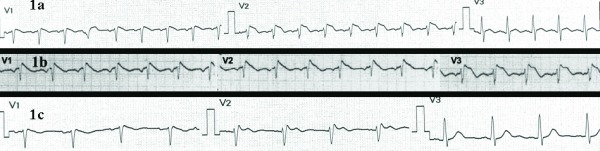
First patient: (a) Admission ECG reveals the typical features of type 1 Brugada pattern with a coved ST-segment elevation with a downsloping ST segment in V1 and V2, associated T wave inversion and pseudo-right bundle branch block pattern, (b) ECG after provocative procainamide test two years ago was similar to the admission ECG, (c) Evolution of Brugada type 1 to type 2 after electrical stabilization.

On admission, the patient was on a febrile status due to pneumonia. All laboratory tests were unremarkable. The ECG revealed the typical features of type 1 BS pattern (Figure 1a) that was similar to the ECG after the procainamide test two years ago (Figure 1b). The patient was started on oral hydroquinidine hydrochloride (600 mg twice daily), antibiotics and anti-fever medication. He remained electrically stable for the rest of his hospitalization. Of note, on the fourth day of hospitalization the characteristic type 1 BS pattern evolved to type 2 (Figure 1c). Hydroquinidine was discontinued on the seventh day and the patient was discharged on good clinical condition. After nine months of follow-up the patient remained stable without any recorded ICD therapy.

## Case 2

**Figure 2:**
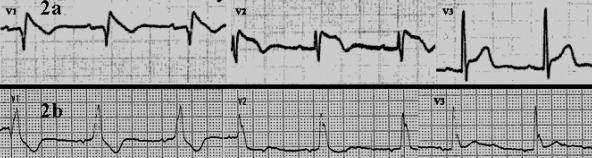
Second patient: (a) Admission ECG demonstrating a type 1 Brugada pattern with coved ST-segment elevation of 3.0 mm with a downsloping ST segment in V1 and V2 and associated T wave inversion, (b) This pattern was resolved five days later with the occurrence of a new onset right bundle branch block.

A 75-year-old man with BS, who was treated with ICD implantation nine years ago due to recurrent syncope and a subsequent positive procainamide test, was admitted for seven episodes of ventricular fibrillation within 24 hours that was successfully treated with appropriate ICD shocks. Since the ICD implantation the patient did not receive any medication. There was no predisposing factor that could lead to the electrical instability. All laboratory tests were unremarkable. The admission ECG had the typical features of type 1 BS pattern (Figure 2a) that was resolved five days later following electrical stabilization with the occurence of a new onset right bundle branch block (Figure 2b).

The patient was started on oral hydroquinidine (600 mg twice daily), remaining electrical stable for the rest of his hospitalization. After six months of hydroquinidine treatment the patient was also asymptomatic without any recorded ICD therapy.

## Discussion

BS is a primary electrical disorder and it is responsible for 20-50% of sudden cardiac deaths in patients with structurally normal hearts [1]. At the molecular level the syndrome is associated with dysfunction of sodium channels which provokes electrical dysfunction, the characteristic ECG pattern and development of arrhythmias. Of note, patients with suspected BS or family mutation carriers may have normal ECG. Diagnostic provocation with a sodium channel blocker, such as ajmaline or procainamide, may usually unmask the BS pattern, verifying the diagnosis [1]. In BS, the prevalence of malignant ventricular arrhythmias varies from 5% in asymptomatic patients to 45% in patients with previous cardiac arrest [2]. Electrical storm may be the first manifestation of the disease. Unfortunately, it is not accurate predictable which patients with BS are more prone to develop electrical storm due to the absence of specific clinical, laboratory, electrocardiographic, and electrophysiologic characteristics associated with electrical storm [3]. Fragmented-QRS, late potentials and T-wave alternans have been proposed as markers for the substrate for spontaneous VF in BS but with limited use in daily clinical practice. [4] Despite our first patient's variations of BS pattern from type 2 to type 1 (Figure 1) under the existence of a precipitating factor, the BS ECG pattern does not seem to be a predictor of outcome in BS patients [5].

Patient with VT/VF storm should be recommended for ICD implant because none of the antiarrhythmic drug therapy reliably prevents sudden cardiac death. Both our patients had already been treated with ICD implantation. With regard to the treatment of electrical storm in BS the contemporary guidelines suggest isoprenaline and quinidine [IIa and IIb class (level of evidence: C)], respectively [6]. Isoprenaline, a β-adrenergic stimulator, increase I_CaL_ function, restoring the dome of epicardial action potential and with this mechanism suppresses electrical storm and reduces the typical ST elevation [7]. Quinidine, a class IA antiarrhythmic agent, blocks the I_to_-channel counteracting the marked abbreviation of action potentials in epicardial cells and thus normalizing the ECG pattern and ventricular excitability [8].

In our cases hydroquinidine was administered for only 7 days in the first patient and suppressed the storm, whereas in the second patient the quinidine was continued for 6 months contributing to the suppression of the electrical storm and long-term prevention of ventricular arrhythmias. Our decision to use hydroquinidine (instead of isoprenaline) for the suppression of the electrical storm was based on the ability to administer the drug per os, making it easier for long-term use.

Recently, a discussion was initiated among electrophysiologists concerning quinidine shortage in the drug market [9]. Quinidine is effective medical treatment for patients with short QT syndrome, BS and a subgroup of idiopathic ventricular fibrillation [9]. Electrical storms in patients with the above syndromes should be treated with ICD. However, since ICD does not prevent the occurrence of arrhythmias, oral quinidine could be a reasonable choice for long-term prevention of life-threatening tachyarrhythmias.
